# Using 42 CFR part 2 revisions to integrate substance use disorder treatment information into electronic health records at a safety net health system

**DOI:** 10.1186/s13722-024-00477-3

**Published:** 2024-06-07

**Authors:** Alexandra R. Tillman, Emily Bacon, Brooke Bender, Dean McEwen, Joshua Blum, Matthew Hoag, Kenneth A. Scott, Rachel Everhart, Rebecca Hanratty, Laura J. Podewils, Carolina Close, John Mills, Arthur J. Davidson

**Affiliations:** Denver Health and Hospital Authority, 777 Bannock Street, Denver, CO 80204 USA

**Keywords:** Substance use disorder treatment, Electronic health record, Hub-and-spoke, Data integration, 42 CFR Part 2

## Abstract

**Background:**

Regulations put in place to protect the privacy of individuals receiving substance use disorder (SUD) treatment have resulted in an unintended consequence of siloed SUD treatment and referral information outside of the integrated electronic health record (EHR). Recent revisions to these regulations have opened the door to data integration, which creates opportunities for enhanced patient care and more efficient workflows. We report on the experience of one safety-net hospital system integrating SUD treatment data into the EHR.

**Methods:**

SUD treatment and referral information was integrated from siloed systems into the EHR through the implementation of a referral order, treatment episode definition, and referral and episode-related tools for addiction therapists and other clinicians. Integration was evaluated by monitoring SUD treatment episode characteristics, patient characteristics, referral linkage, and treatment episode retention before and after integration. Satisfaction of end-users with the new tools was evaluated through a survey of addiction therapists.

**Results:**

After integration, three more SUD treatment programs were represented in the EHR. This increased the number of patients that could be tracked as initiating SUD treatment by 250%, from 562 before to 1,411 after integration. After integration, overall referral linkage declined (74% vs. 48%) and treatment episode retention at 90-days was higher (45% vs. 74%). Addiction therapists appreciated the efficiency of having all SUD treatment information in the EHR but did not find that the tools provided a large time savings shortly after integration.

**Conclusions:**

Integration of SUD treatment program data into the EHR facilitated both care coordination in patient treatment and quality improvement initiatives for treatment programs. Referral linkage and retention rates were likely modified by a broader capture of patients and changed outcome definition criteria. Greater preparatory workflow analysis may decrease initial end-user burden. Integration of siloed data, made possible given revised regulations, is essential to an efficient hub-and-spoke model of care, which must standardize and coordinate patient care across multiple clinics and departments.

**Supplementary Information:**

The online version contains supplementary material available at 10.1186/s13722-024-00477-3.

## Background

Challenges engaging and effectively treating patients experiencing substance use disorder (SUD) are deep and broad; those barriers contributed to 107,622 overdose deaths in the United States in 2021 [1]. Approximately 65% of overdose deaths in 2022 had a documented missed opportunity for intervention at the time of death [2]. A steady increase of overdoses and related deaths has resulted in more focused treatment and recovery services funded and organized by federal agencies [3].

When treatment services are established in a separate physical or administrative location from usual healthcare, information siloes develop [4]. Federal regulations for federally funded SUD treatment programs under Title 42 Consolidated Federal Register part 2 (42 CFR part 2) [5] were designed to protect patient privacy (e.g., avoid residual stigma, labeling or non-medical access to records); an unintended consequence has been interference with care integration. Integration of care has improved chronic disease management [6]. In a systematic review of primary care treatment of opioid use disorder (OUD), electronic health records (EHR) and data sharing were identified as facilitating better communication and integrated care across multidisciplinary treatment teams [7]. However, communication and integration are often restricted when a patient has received SUD treatment in a federally assisted (“part 2”) program. Even within integrated health care entities, compliance and legal advice frequently have focused on risk-aversive postures, such as documentation outside of the EHR for integrated SUD care [8]. Organizations, like California Health Care Foundation, have recommended solutions, specifically for SUD [9] which highlighted standardized consent training, forms, and processes to promote greater SUD health information exchange. Similarly, the Substance Abuse and Mental Health Services Administration (SAMHSA) and the Office of the National Coordinator for Health Information Technology have encouraged use of directed and/or query-based health information exchange [8]; theoretical scenarios illustrate how a revised Part 2 applies to patient health information disclosures.

Additional challenges exist for patients with a SUD given adverse social determinants of health are associated with more severe SUD [10]. Patients with a SUD who experience stigma and fear are marginalized, often receiving chaotic or disjointed care across multiple health care venues [11]. Safety net institutions [12] are key service providers to communities with limited access to healthcare and socioeconomic resources; these essential providers offer evidence-based SUD care (e.g., medications for SUD and ancillary services) at reduced cost. Since 2010, safety-net institutions were incentivized to adopt, implement, and update certified EHR technology [13]. These advances increased capacity for building EHR disease registries (e.g., diabetes or SUD). While diabetes registry development was fairly straightforward, given privacy rules, SUD treatment data (compliant with 42 CFR part 2) have traditionally been stored in physically or virtually separate systems, sometimes external to the EHR, often making coordinated treatment and program evaluation impossible. This patchwork of health care and confidentiality regulations has reinforced siloed information, care fragmentation and poor engagement, impeding more integrated, patient-centered care.

Recent models of SUD care have defined more effective ways to engage patients, like the “hub and spoke” model [14] which engage patients where they present and then assess if they are more appropriate for treatment at a “hub” (licensed specialty outpatient treatment programs with ability to dispense methadone and buprenorphine) or a “spoke” (outpatient medical practices that can provide office-based opioid treatment with buprenorphine). Additionally, there has also been greater adoption of a continuum of care [15, 16] model to identify gaps in various stages of engagement, treatment and recovery of SUD. The path through the health system is frequently non-linear for patients seeking recovery. Any certified EHR has the technical capacity to support greater communication between individuals, groups, and organizations who comprise the patient’s health care team [17]. Integrated EHR communication has reduced pediatric trauma length of stay [18], reduced costs and patient safety events (i.e., medication safety [19]), improved quality measures with more documentation through standardized health processes [20], and documented needs for cross-program communication (e.g., mental health or health-related social needs) needed for effective individual treatment (SUD or otherwise [21]). Secure EHR data sharing is being encouraged through federal reimbursement programs [22]. With enhanced data sharing, programs would improve patient-centeredness and more accurately track progression along a care continuum and better serve those in transition (e.g., justice-involved [23]).

The 2020 revision of 42 CFR part 2 [24] has offered a regulatory opportunity for enhanced data sharing. Explicit consenting processes now exist for patients to disclose their SUD-related information for operational purposes without naming a specific care team individual, allowing SUD treatment information to be visible within the EHR across the health system. Combined with the advent and near ubiquity of EHR systems across the health care ecosystem [25], new opportunities for data sharing are possible. Given this revised 42 CFR part 2 regulation, safety-net institutions frequently implementing hub and spoke models of care [26, 27] may be prime locations for testing this new data sharing paradigm. We share our experience implementing and evaluating the integration of siloed SUD treatment data in a hub and spoke care model in the EHR at one safety-net institution.

## Methods

### Setting

Denver Health (DH), a large, integrated, public safety-net healthcare system in Colorado [28], provides emergency medical services, inpatient treatment in a 500-bed hospital, outpatient primary and specialty care across 10 federally-qualified health centers (FQHCs) and 19 school-based clinics, and federally funded substance treatment services. DH serves approximately 250,000 patients annually across its facilities. In 2017, while there was no comprehensive registry for SUD, a cross-sectional continuum of care analysis [16] identified 3,300 individuals with OUD at DH. Using diagnosis codes and additional markers of OUD identified in the 2017 continuum of care analysis [16], an internal analysis estimated that nearly 11% of patients served by DH in 2022 had some kind of SUD (unpublished observations, ART).

Since 2016, DH has utilized a certified EHR (Epic, Verona, WI, USA) to document patient care. Parallel to the EHR, a separate set of spreadsheets and databases have been developed and maintained over decades to comply with 42 CFR part 2. These legacy and standalone systems required special access and authorization. In 2020, an updated DH consent for the use and disclosure of substance use and treatment information was revised to say that specialty SUD treatment data could be shared across the system with any DH provider involved in the patient’s care, designating all of DH specialty SUD treatment programs as one entity under 42 CFR part 2.

As informed by SUD treatment care team members anecdotally and through an internal needs assessment (which included thematic analysis of structured key informant interviews and focus groups with staff), tracking and/or communicating with other care team members about a patient were challenging tasks. To see a comprehensive patient story required labor intensive review of provider notes across multiple data sources (e.g., programs), and time (e.g., episodes [29]). Unstructured provider documentation precluded efficient outcome evaluation for individuals or populations. Addiction therapists redocumented information into a separate spreadsheet to track tasks and outcomes. Rather than an EHR-mediated referral, messaging started through email or a telephone call with an addiction therapist, who maintained their own spreadsheet for tracking. Caseload information was stored in a separate database which hampered ready access by supervisors for monitoring. A standard, cross-program definition for SUD treatment episodes was needed.

**Fig. 1:**
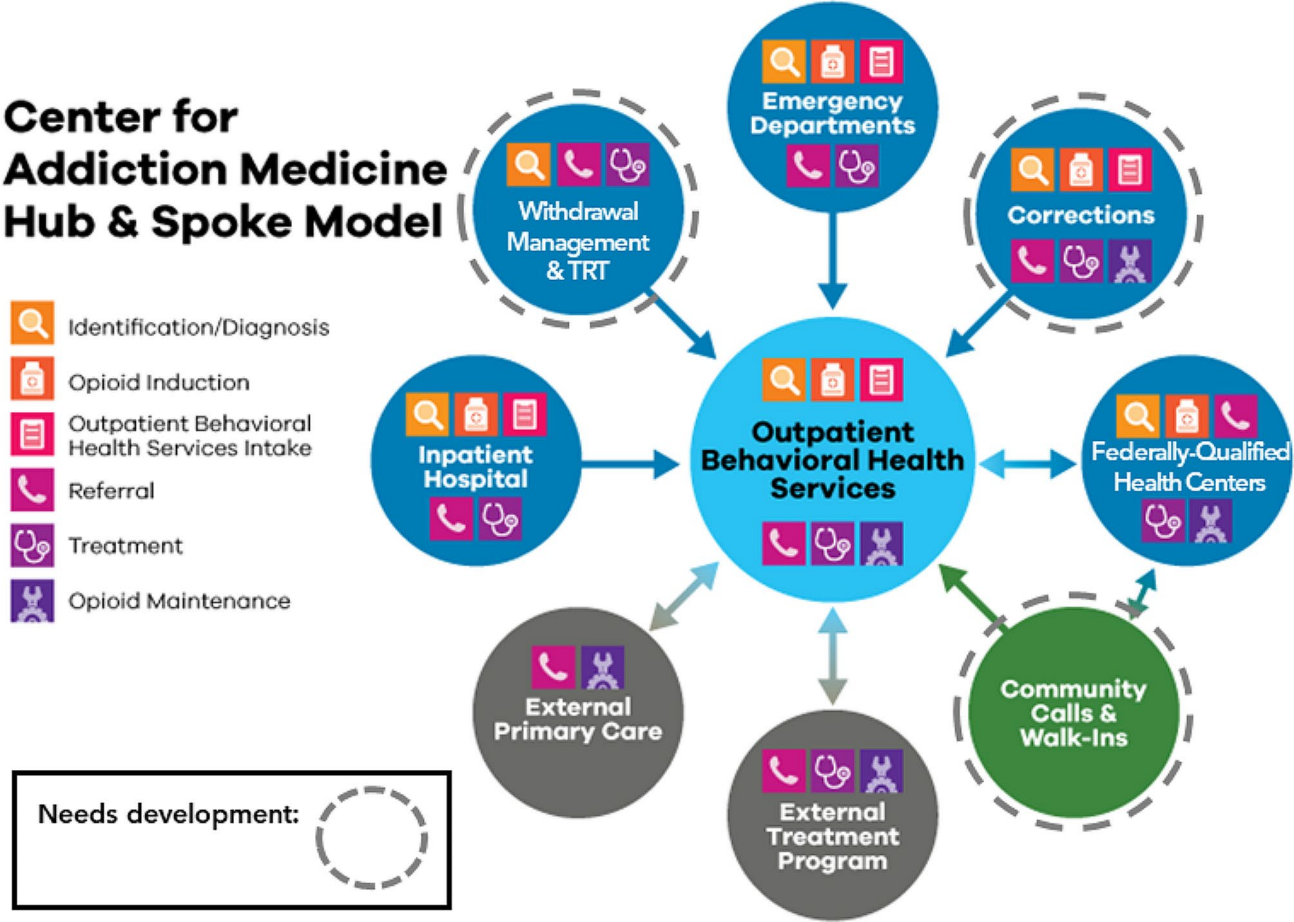
Hub and Spoke Model diagram for integrating substance use disorder engagement, treatment, and recovery care from multiple entry points, Denver Health, Denver CO, 2023

In 2019, DH established the Center for Addiction Medicine (CAM) to provide the infrastructure to integrate SUD care employing a hub and spoke model (see Fig. 1), different from traditional models that span multiple health systems and are state-wide (e.g. Vermont [30], Washington [14]). A hub resides in Outpatient Behavioral Health Services (OBHS), which provides specialty outpatient addiction services, including methadone treatment and care for priority populations including pregnant women and adolescents. Spokes include referral sources to the hub including an inpatient addiction consult service; emergency services, with 24/7 opioid agonist induction and linkage to care; and FQHCs providing integrated primary care with co-located SUD addiction therapists and behavioral health professionals. Outpatient SUD treatment occurs at FQHCs and several OBHS programs: (1) a general office-based addictions clinic, (2) an OUD dispensary clinic, (3) an adolescent SUD clinic, and (4) specialty women and family SUD services. Since its inception, CAM has provided the infrastructure, resources, and human capital to develop workflows to move patients from spokes to hubs. All of these locations utilize a single EHR instance which is managed and deployed by the DH information technology department. Areas still targeted for future data integration (stippled circles in Fig. 1) represent the withdrawal management service, the transitional residential treatment program, calls to the community line, and correctional care. The DH CAM hub and spoke model also includes strong partnerships with local organizations that provide residential treatment and other levels of care that DH does not offer internally.

### EHR data integration enhancements

To establish standardized and streamlined SUD processes for referral and treatment episode tracking, two categories of EHR enhancements were implemented. The first was a treatment referral order that replaced phone calls, emails, and spreadsheets previously employed for making and tracking referrals from inpatient and ED locations. The new SUD treatment referral order, a single customized EHR referral order, collected patient-specific data from inpatient and ED SUD counselors to ensure the necessary information was available when the patient presented for treatment at the location receiving the referral. The second set of enhancements was a standardized treatment episode definition and episode-related tools that replaced program-specific definitions and data capture external to the EHR. Episodes of care needed a uniform definition for data sharing between programs (hub and spokes). Clinical and CAM leaders came together and decided that the new standardized treatment episode definition would start when an addiction therapist completes a comprehensive (1–1.5 h) biopsychosocial intake (which occurs in OBHS, FQHCs, ED, and inpatient setting) and ending on the discharge date from outpatient services. The new EHR-defined SUD treatment episode allowed standard information to reside within the patient’s chart, eliminating redundant documentation and expanding standardized documentation to additional existing programs. Episode tools customized to the new SUD treatment episode allowed users to easily manage standard episode-level information such as substance(s) of use and primary addiction therapist. Tools were centralized in an EHR-based dashboard where users were able to monitor SUD treatment checklists and oversee caseloads, among other capabilities, instead of in an external Microsoft Access database as had been done prior to enhancement.

**Fig. 2:**
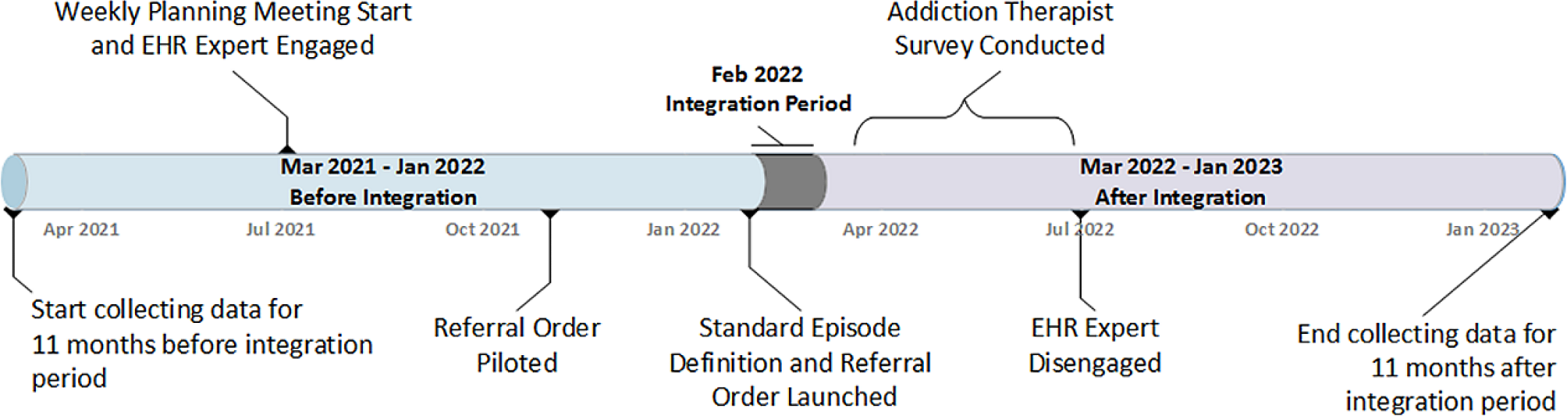
Timeline for electronic health record data integration, March 2021 – January 2023. Denver Health, Denver CO.

Implementation of these EHR data integration enhancements began in earnest in July 2021. At this time, DH engaged an EHR consultant to efficiently design and develop data integration and maintenance enhancements. The EHR consultant began with weekly planning and feedback meetings with CAM team leadership (i.e., epidemiologist and public health planner) and clinical leadership. Clinical leadership contributed to the development of the system and defined integration requirements. The SUD treatment referral order was piloted in November 2021 and the SUD treatment episode definition and tool were piloted in a single FQHC clinic in Summer 2021. All tools were fully launched in February 2022. Data from external systems were imported to establish episodes for active patients right before the launch. A one-month, post-launch period allowed for training and uptake before post-implementation evaluation data collection. All day support was available for end-users via a communications tool (Webex) for the first week of operations. Ongoing weekly meetings were conducted with the EHR consultant, clinical and CAM leadership to assess enhancement use, identify issues, and provide feedback to the development team. By the third quarter of 2022, periodicity of meetings decreased to quarterly. Figure 2 represents a timeline of integration activities. Before integration (between March 1, 2021 and January 31, 2022), SUD treatment data were compiled monthly from systems external to the EHR from only one of four OBHS programs as it was the only program collecting SUD treatment initiation data and referral data from ED and inpatient areas. After integration (between March 1, 2022 and January 31, 2023), all previously existing OBHS and FQHC programs contributed standardized data into the common EHR, along with referral orders from ED and inpatient areas.

### Evaluating the impact of integration

We evaluated the impact of EHR integration by comparing multiple factors before and after integration. Factors included (1) SUD treatment referral and episode volume; (2) patient characteristics; (3) treatment episode characteristics; (4) linked referrals; (5) treatment episode retention; and (6) tool satisfaction.

*SUD treatment referral and episode volume*: The number of referrals from ED and inpatient areas as well as SUD treatment episodes started within the before and after integration periods were collected and compared.

*Patient characteristics*: Patients undergoing SUD treatment before and after integration were compared on sociodemographic factors including sex, age, race/ethnicity, insurance class, and housing status, which were collected from the EHR. A patient was considered unhoused if there was evidence in the EHR that the patient was unhoused at any point in the observation period.

*Treatment episode characteristics*: The department where treatment was initiated and the primary substance of SUD treatment episodes were also compared before and after integration to understand whether types of SUD treatment episodes changed after integration. Patients could fall out of care and re-enter the process multiple times and in either period, each of which was considered a distinct episode of care.

*Linked referrals*: Referral linkage was tracked before and after integration. Integrating data in the EHR meant creating one common, operational definition of linkage. Therefore, linkage definitions were different before and after integration. Before integration, linkage was defined for those with OUD as a medication for opioid use disorder (MOUD) dose in the outpatient setting. Linkage was defined for those with a SUD (other than OUD) as an outpatient counseling visit after a referral from a hospital stay or ED visit. After integration, linkage was defined as whether a patient referred to SUD treatment from the hospital or ED had a completed appointment at an outpatient clinic (OBHS or FQHC) within 30 days of discharge. Before integration, referral data were collected from an external system and matched to the EHR to capture outpatient treatment within 30 days of the treatment referral. After integration, linkage had the same definition for all SUDs and data on referral and completed outpatient appointment after referral were collected entirely from the EHR.

*Treatment Episode retention*: 90-day retention in SUD treatment episodes was defined as whether a patient was retained in SUD treatment for 90 days or more. Similar to linkage, the definition of retention was standardized as part of integration. For both periods, episode start dates were the date the biopsychosocial intake was completed by an addiction therapist. Before integration, patient discharge information was not reliably recorded, which meant that the only reliable definition of retention in care was based on continuation of MOUD (for patients with OUD) or continuation of counseling services (for patients with a SUD other than OUD) without gaps in treatment. After integration, per the new SUD treatment episode definition, episode end dates were outpatient treatment program discharge dates.

*Tool satisfaction*: To ensure the system was developed as intended, we conducted a 17-question survey (supplemental information) among addiction therapists, developed from existing standardized surveys [31, 32], focused on whether the data integration efforts improved: continuity of care, efficiency of placing referrals, quality of care, patient monitoring, sharing of information, personal efficiency, and patient safety. The survey also asked about ease of analyzing outcomes of care, communicating with colleagues to coordinate care, and facilitating care documentation. The survey used a combination of Likert scale responses and open-ended questions. Requests to complete online surveys were sent to addiction therapists across departments (i.e., OBHS, FQHCs, ED, and Inpatient) in May 2022, three months after EHR integration tools were deployed. Respondents completed the survey online and data were collected and managed using REDCap electronic data capture tools [33]. Survey response rate was stratified by department. Questions were tabulated overall and by department. Open ended questions were grouped by department and coded for common themes.

Descriptive analyses of quantitative metrics and chi-square tests of categorical patient characteristics were carried out using SAS Enterprise Guide software, Version 8.3 of the SAS System for Windows (SAS Institute Inc., Cary, NC, USA). Survey results were analyzed in Tableau Desktop (Tableau, Seattle, WA, USA).

## Results

*SUD treatment referral and episode volume*: Within DH, a total of 1,892 patients with SUD had 2,110 treatment initiations documented in the EHR and parallel information system during the two observation periods. Before integration, 562 patients were documented as having initiated SUD treatment. After integration, 1,411, or 250% as many patients, were documented as having initiated SUD treatment, compared to the period before integration.

*Patient characteristics*: There were age differences before and after integration (chi-square *p* < 0.01). When stratified by age categories (see Table 1), an increase in younger patients 12–17 years old after integration was likely due to inclusion of episode documentation from the existing adolescent treatment program at OBHS. Additional patients, as well, were observed at older ages (56–65 years) after integration. Comparing before and after integration, the racial makeup of patients was different (chi-square *p* = 0.04). The proportion of non-White patients with a SUD treatment episode was marginally lower after integration. A slight change in insurance types at treatment initiation was also significant (chi-square *p* = 0.03); fewer patients were on public insurance options after integration. The percentage of unhoused patients was lower after integration (chi-square *p* < 0.01).

**Table 1:** Demographic and clinical characteristics of individuals initiating substance use disorder treatment, before (March 2021 – January 2022) and after (March 2022 – January 2023) an electronic health record data integration project, Denver Health, Denver CO.

	Time Period: Relative to Electronic Health Record Data Integration				Chi-square *p*-value
**Characteristics**	Before		After		
	*N* = 562	(%)	*N* = 1411	(%)	
**Sex**					0.75
Female	198	(35)	508	(36)	
Male	364	(65)	903	(64)	
**Age (years)**					< 0.01
12–17	4	(< 1)	140	(10)	
18–25	60	(11)	128	(9)	
26–35	232	(41)	426	(30)	
36–45	155	(28)	403	(29)	
46–55	77	(14)	173	(12)	
56–65	25	(4)	122	(9)	
> 65	9	(2)	19	(1)	
**Race/ethnicity**					0.04
Non-Hispanic White or Caucasian	294	(52)	661	(47)	
Hispanic	205	(36)	540	(38)	
Non-Hispanic Black or African American	42	(7)	121	(9)	
Multiracial/Other/Unknown	21	(4)	89	(6)	
**Insurance class at treatment initiation**					0.03
Commercial	32	(6)	124	(9)	
Public (Medicaid and/or Medicare)	478	(85)	1118	(79)	
Uninsured	17	(3)	53	(4)	
Other/Unknown	35	(6)	116	(8)	
**Housing status at any point in period**					< 0.01
Unhoused	232	(41)	439	(31)	
Housed	330	(59)	972	(69)	

*Treatment episode characteristics*: Information on the department where treatment was initiated and primary type of SUD are described in Table 2. Integration of data from additional programs at specific departments was evident from after integration as compared to before. There was also a noteworthy change in the distribution of self-described primary SUD. While OUD was the overwhelming primary SUD before integration (91%), after integration there was a large increase in those who identified alcohol (+ 19%), cannabis (+ 7%), and stimulant use (+ 3%) as their primary SUD due primarily to additional non-opioid specific programs contributing data after integration.

**Table 2:** Treatment initiation department, and primary substance use disorder type for substance use disorder treatment episodes, before (March 2021 - January 2022) and after (March 2022 - January 2023) an electronic health record data integration project, Denver Health, Denver CO.

	Time Period: Relative to Electronic Health Record Data Integration			
**Characteristic**	**Before**		**After**	
	*N* = 608	(%)	*N* = 1502	(%)
**Treatment initiation department**				
Community Health Service	0		257	(17)
Outpatient Behavioral Health Service	194	(32)	699	(47)
Emergency Department	246	(40)	281	(19)
Inpatient	168	(28)	265	(18)
**Primary substance use disorder type**				
Alcohol	40	(7)	398	(26)
Cannabis	0		104	(7)
Opioids	552	(91)	900	(60)
Other	0		14	(< 1)
Stimulants	16	(3)	86	(6)

*Linked referrals*: After integration, the definition of linkage to care was the same for all SUDs and extracted from the EHR. The change in the linkage definition impacted linkage results after integration to more accurately capture patient linkage to outpatient care. Linkage to outpatient care after integration was 26% points lower for all SUD types compared to the period before integration (Table 3).

**Table 3:** Substance use disorder linkage to care and 90-day treatment retention outcomes, by primary substance use disorder type, before (March 2021 - January 2022) and after (March 2022 - January 2023) an electronic health record data integration project, Denver Health, Denver CO.

	Time Period: Relative to Electronic Health Record Data Integration				Difference in Percentage Points
Substance Use Disorder Type	Before		After		
	Numerator / Denominator	%	Numerator / Denominator	%	
**Linked referrals / Total referrals**					
All Substance Use Disorders †	272 / 369	74	233 / 489	48	-26
Alcohol Use Disorder	19 / 20	95	34 / 60	57	-38
Opioid Use Disorder	210 / 342	61	232 / 414	56	-5
Stimulant Use Disorder	5 / 7	71	4 / 13	31	-40
**Episodes retained 90 days / Total episodes**					
All Substance Use Disorders †	190 / 420	45	913 / 1228	74	29
Alcohol Use Disorder	9 / 25	23	280 / 328	85	62
Opioid Use Disorder	177 / 386	46	483 / 710	68	22
Stimulant Use Disorder	4 / 9	44	58 / 73	79	35

† While All Substance Use Disorders includes Cannabis Use Disorder and Other Use Disorder episodes and referrals, those specific data were not disaggregated and presented due to small numbers; thus, these totals exceed the sum of substance use disorders represented.

*Episode retention*: Retention of at least 90 days in outpatient care, which was the outcome of episodes that entered the outpatient setting, was higher after integration for all SUD types compared to the period before integration. Results in Table 3 show that 74% of patients were retained in care after 90 days using the new retention definition after integration.

*Tool satisfaction*: Results from the survey of addiction therapists provided insights into how primary users perceived the EHR data integrated enhancements. Of the 38 addiction therapists across all programs, 26 completed the survey. The overall response rate was 68%, with the Inpatient/ED group reaching 3 of 5 (60%), FQHCs reaching 8 of 9 (88%), and OBHS reaching 15 of 24 (63%).

Responses to open-ended questions demonstrated that respondents across departments appreciated the ability to have everything in the EHR rather than in separate systems. Other benefits of the integration and areas left for improvement differed by therapist department (Table 4). FQHC therapists valued the user-interface in the EHR they were accustomed to working in, but found they were doing more work to document than the prior state (as these therapists were not keeping separate caseload spreadsheets); OBHS therapists liked that they could see when a patient was admitted to the DH hospital and their caseload information from the EHR dashboard, but would have preferred more training in the roll out; ED/Inpatient therapists appreciated pre-populated choices in the referral order form, but wanted to be able to visualize if their patients linked to care beyond the DH system.

**Table 4:** Qualitative themes from tool satisfaction survey of addiction therapists by department after an electronic health record data integration project, Denver Health, Denver CO.

	Outpatient Behavioral Health Services (OBHS)	Federally Qualified Health Centers (FQHCs)	Emergency Department (ED)/Inpatient
Benefits appreciated by end-users	- Referral/caseload info in EHR - Easy to understand missing/overdue care tasks - Ability to see when patient is admitted to hospital - Visibility of caseloads on dashboard in EHR	- Referral/caseload info in EHR - User friendly interface within EHR	- Referral/caseload info in EHR - Prepopulated referral choices
Areas for improvement identified by end-users	- More training - No initial time savings	- Enhancements required more work than prior state - Education to improve workflows	- Cannot visualize patient linkage beyond DH internal clinics

## Discussion

The 2020 revision of 42 CFR part 2 permits inclusion of data derived from federally funded substance use treatment programs to support care coordination. The revisions permitted integration of multiple independent, SUD-related data sources, within a single EHR; comparing patient characteristics before and after this data integration, many more health system patients with SUDs, including opioids, were identified. This integration required new EHR workflow enhancements including a standardized treatment referral order, and a standardized treatment episode definition and episode-related tools. The new infrastructure supported a “hub and spoke” model of coordinated SUD treatment and care, at a large safety-net healthcare system.

To retrieve EHR data about patients from all areas of the health system required changing the operational definition of linkage to care to include *all* patients referred to SUD treatment; all SUD treatment referrals (i.e., from hospital, ED) were considered linked to care if within 30 days of discharge the patient completed an outpatient SUD treatment appointment, rather than just patients with OUD receiving an MOUD dose in the outpatient setting. Likely because of this changed definition and a more comprehensive patient denominator, linkage rates were lower after integration. The definition of retention in care after 90 days also changed after EHR integration to reflect program discharge dates within a specific episode of care instead of continuation without 30-day breaks in MOUD dispenses or counseling services. Patients interact with SUD treatment services in diverse ways that should be considered retained, which are not easy to define from traditional administrative data sources (like encounters with therapists or medication dispenses). Therefore, it was important to use the discharge date from care to more accurately reflect when a program considered a patient no longer retained. Driven by broadening the outcome definition and potentially from a more inclusive patient base, 90-day retention appeared higher after integration compared to before.

Implementing new standardized definitions of episodes, linkage, and retention in care allowed for a more complete enumeration of patients utilizing SUD treatment, across the health system. Although before and after integration metrics are not directly comparable, differences in linkage and retention suggested an expanded definition included more patients in standardized SUD tracking, which going forward will be more efficiently and meaningfully be tracked. This sentiment was captured in the survey of addiction therapists using the new tools. Many said the new tools permitted a broader view of SUD and its treatment across the healthcare system.

In addition to more streamlined clinical processes, the integrated EHR enhancements support both treatment for and surveillance of a spectrum of programs for patients with SUD. New SUD Tableau dashboards, based on EHR data, leveraged the common episode definition and data collection processes to monitor treatment initiation and episodes of care, across all programs after integration. A benefit of this data integration project has been its extensibility beyond opioid use disorder. The final design has enabled a substance-agnostic technical solution to address broader SUD treatment and care needs for the safety-net healthcare population. Systemwide analyses now include any patient or population with SUD, including their stage along the continuum of care. Epidemiologic subpopulation analyses permit better visualization of variable treatment success for specific groups to target interventions. These linkage and retention metrics align with newly-established metrics from the Centers for Disease Control and Prevention Overdose Data to Action Initiative [34]. Standardized definitions for health care providers as well as states and local health departments will allow for more efficient national tracking of SUD treatment processes, especially for specific populations. Those populations of interest are often based on specific entry points to care. CDC-defined priority entry points include the ED, emergency medical services, other clinical settings, criminal justice, harm reduction programs, self-referrals, or other community-based programs. The data dashboards developed with the data made available by this project are capable of tracking these precise measures, stratified by entry point, with feedback loops for quality improvement. A strength of this approach was the active involvement from end users, including addiction therapists and clinicians who manage SUD. Building worthwhile EHR enhancements for SUD program staff required their time, providing feedback to EHR specialists during design and testing of program modifications and software implementation. Initially, data integration enhancements saved therapists no time, as they input the same, if not more, data into the EHR.

As of February 8, 2024 after the rollout of these integrated SUD tools, the U.S. Department of Health & Human Services released the final rule modifying 42 CFR Part 2 [35]. The final rule implements the 2020 revision of 42 CFR Part 2 which aligns consenting and disclosure practices with the Health Insurance Portability and Accountability Act. Having integrated tools within the EHR should position Denver Health to implement the new rule much more efficiently than prior siloed systems. Once an updated consent is obtained electronically, SUD data may be shared with a broader group of internal and external departments, which further streamlines SUD care. In addition to the new 42 CFR Part 2 final rule, other efforts should increase standardization and interoperability of SUD treatment. County level efforts should be possible with more standardized definitions [34], especially with data sharing as proposed in (1) recent federal funding toward Qualified Health Information Networks [36, 37] that promote greater interoperability and (2) the 42 CFR Part 2 final rule [35] with greater emphasis on care coordination and consent-based sharing of SUD treatment data. Establishing governance needed for multi-agency efforts is non-trivial; engaging in that work early will yield benefit as this work has highlighted.

When integrating data, frontline program staff saw value in their ability to support patients more comprehensively. In retrospect, as the project evolved it was clear we needed much more front-line staff input during development, implementation, and operations. While executive sponsorship and leadership were crucial to get the project underway, these workflows were much better understood and informed by the addiction therapists themselves.

A limitation of before/after EHR data integration project assessments is uncontrolled and parallel changes to data systems and programs; specific attribution of change in key outcomes (e.g., referrals linked to outpatient treatment and episodes retained in outpatient care) is difficult. Lower rates of linkage to outpatient treatment after integration was most likely attributable to standardizing how linkage was measured and more complete patient enumeration rather than less program success in linking patients. Challenges were observed in acquiring all data needed for a fully implemented hub and spoke model. Some hub-and-spoke data partners were not ready to resolve operational, technical and governance issues for sharing their program’s data, especially those beyond the health care environment (see Fig. 1, stippled circles). Even in health care, an individual who inquired through a “community call-in” would not be established as a patient in the EHR until a visit has been made. The county jail had no EHR, functionally hindering its participation. Patients entering DH’s withdrawal management program are at times involuntarily entered into the program by police; these data are under extra protections as consent cannot be obtained. No single study would be able to definitively resolve all technical and governance issues related to optimal data integration. We described the process, results, benefits, and challenges of developing a more coordinated and patient-centered approach. After years of limited data sharing based on federal rules, we have more intently begun this journey.

Future improvements and planning have focused on training end-users to benefit more from the additional functionalities of integrated systems. New data analyses should focus on further operationalizing the continuum of care [16] and its use as a resource to inform outreach and referral to services required to address their social determinant of health needs. The 42 CFR Part 2 final rule [35] permits (with consent) DH (and many other safety-net health care providers) to securely exchange SUD referral and treatment data with community partners who support a spectrum of social issues for these patients as they seek healthier lives.

## Conclusion

Our experience highlights how integration of SUD treatment program data into the EHR can facilitate both care coordination in treatment of patients with SUD and monitoring of 42 CFR part 2 programs for quality improvement initiatives. A hub and spoke model has been initially operationalized with inputs from multiple entry points across one system; other health care systems can use these lessons learned to leverage their own data integration efforts to support SUD engagement, treatment, and recovery services within their communities.

### Electronic supplementary material

Below is the link to the electronic supplementary material.

Supplementary Material 1

## Data Availability

The data that support the findings of this study are not openly available due to reasons of sensitivity and are available from the corresponding author upon reasonable request. Data are located in controlled access data storage at Denver Health and Hospital Authority.

## Abbreviations

42 CFR Part 2: Title 42 Consolidated Federal Register part 2
CAM: Center for Addiction Medicine
DH: Denver Health
ED: Emergency Department
EHR: Electronic Health Record
FQHC: Federally-Qualified Health Center
MOUD: Medications for Opioid Use Disorder
OBHS: Outpatient Behavioral Health Services
OUD: Opioid Use Disorder
SAMHSA: Substance Abuse and Mental Health Services Administration
SUD: Substance Use Disorder
