# Supplementary material for: Using 42 CFR part 2 revisions to integrate substance use disorder treatment information into electronic health records at a safety net health system
Source: Addict Sci Clin Pract. 2024 Jun 7;19:48. doi: 10.1186/s13722-024-00477-3 (PMC11157711; doi:10.1186/s13722-024-00477-3)
Supplement: Supplementary file 1 — Supplementary Material 1 [file 13722_2024_477_MOESM1_ESM.pdf]

April 14, 2022

Substance Use Disorder Treatment Tools in Epic
Implementation Follow-up Survey

Greetings!

The Center for Addiction Medicine (CAM) and **D**ata **A**nalytics to **S**upport the **A**lignment of **H**igh **R**isk Adult Programs and Services (**DASHR**) teams want to learn more about your experience using the new substance use disorder treatment (SUDT) tools in Epic. Your feedback is very important to us. This survey will take about 5 minutes to complete. Responses are confidential. If you have any questions or concerns, please contact Alex Tillman (alexandra.tillman@dhha.org).

Thank you!

The CAM/DASHR Teams

1. Which description below best describes you?

☐ I am a **Community Health Services (CHS) substance treatment counselor** and in Epic I use the SUDT episode and other related tools (e.g., synopsis, dashboard, reports, and PROMIS[1] survey best practice advisory).

☐ I am a **Treatment on Demand (TOD) counselor** and in Epic I use the SUDT referral and other related tools (e.g., reports, hospital discharge notification, episode).

☐ I am an **Outpatient Behavioral Health Services (OBHS) counselor** and in Epic I use the SUDT episode other related tools (e.g., checklist, synopsis, dashboard, reports, and critical incident form).

☐ Other

   a. ☐ Please identify your role and what SUDT tools in Epic you use:

   ____

[1] Patient-Reported Outcomes Measurement Information System

2. This question asks about changes to **patient care and workflows**. Please consider how things were *before* the new SUDT tools were implemented (e.g., tracking in Access database, spreadsheets) and how things are *now* (i.e., all tracking in Epic).

   To what extent do you agree or disagree with each statement below?

**Using the SUDT tools…**	Strongly disagree	Moderately disagree	Moderately agree	Strongly agree	Not applicable to my role
• enhances the continuity of care my organization can provide	◻	◻	◻	◻	◻
• enhances the efficiency of placing referrals	◻	◻	◻	◻	◻
• improves the quality of care I can provide	◻	◻	◻	◻	◻
• improves patient monitoring and follow-up	◻	◻	◻	◻	◻
• improves the sharing of patient information	◻	◻	◻	◻	◻
• increases my efficiency	◻	◻	◻	◻	◻
• reduces risks to patient safety	◻	◻	◻	◻	◻

3. This question asks about changes to your **day-to-day work**. Please consider how things were *before* the new SUDT tools were implemented (e.g., tracking in Access databases, spreadsheets) and how things are *now* (i.e., all tracking in Epic).

   To what extent do you agree or disagree with each statement below?

**Using the SUDT tools has made it *easier* for me to…**	Strongly disagree	Moderately disagree	Moderately agree	Strongly agree	Not applicable to my role
• analyze outcomes of care	☐	☐	☐	☐	☐
• communicate with my colleagues to coordinate care	☐	☐	☐	☐	☐
• document care for my patients	☐	☐	☐	☐	☐
• fulfill reporting requirements	☐	☐	☐	☐	☐
• manage referrals	☐	☐	☐	☐	☐
• obtain and review patient information and data	☐	☐	☐	☐	☐

4. This question asks about your overall **experience and satisfaction** with the new SUDT tools.

   To what extent do you agree or disagree with each statement below?

	Strongly disagree	Moderately disagree	Moderately agree	Strongly agree	Not applicable to my role
• I would recommend the SUDT Tools to other organizations	☐	☐	☐	☐	☐
• Overall, I am satisfied with my experience using the SUDT Tools	☐	☐	☐	☐	☐
• Using the SUDT Tools requires me to do less work compared to what I used to do	☐	☐	☐	☐	☐
• With the SUDT Tools, I now have more time to focus on patient care	☐	☐	☐	☐	☐

5. **What do you like about the new SUDT tools?**

   ____

   ____

6. **What could be improved in the new SUDT tools?**

   ____

   ____

7. Is there anything else that you want to share with us?

____

____

This survey was development in part using the following questionnaires available on AHRQ's Health IT Survey Compendium:

1. Canada Health Infoway System And Use Assessment Survey. This 2015 questionnaire is designed to be completed by administrators, clinical staff, and pharmacists across a health care system. The tool includes questions to assess the usability of clinical decision support systems, electronic health records, and enterprise systems.

2. Office CPO Survey. This 2013 questionnaire is designed to be completed by physicians and clinical staff in a hospital. The tool includes questions to assess usability and attitudes regarding of electronic health records/electronic medical record.
